# Complete Nucleotide Sequence of CTX-M-15-Plasmids from Clinical *Escherichia coli* Isolates: Insertional Events of Transposons and Insertion Sequences

**DOI:** 10.1371/journal.pone.0011202

**Published:** 2010-06-18

**Authors:** Annemieke Smet, Filip Van Nieuwerburgh, Tom T. M. Vandekerckhove, An Martel, Dieter Deforce, Patrick Butaye, Freddy Haesebrouck

**Affiliations:** 1 Department of Pathology, Bacteriology and Avian Diseases, Faculty of Veterinary Medicine, Ghent University, Merelbeke, Belgium; 2 Laboratory of Pharmaceutical Biotechnology, Ghent University, Ghent, Belgium; 3 NXTGNT sequencing facility, Ghent University, Ghent, Belgium; 4 Biobix-Laboratory for Bioinformatics and Computational Genomics, Department of Molecular Biotechnology, Ghent University, Ghent, Belgium; 5 Department of Bacteriology and Immunology, CODA-CERVA-VAR, Brussels, Belgium; National Institute of Allergy and Infectious Diseases, National Institutes of Health, United States of America

## Abstract

**Background:**

CTX-M-producing *Escherichia coli* strains are regarded as major global pathogens.

**Methodology/Principal Findings:**

The nucleotide sequence of three plasmids (pEC_B24: 73801-bp; pEC_L8: 118525-bp and pEC_L46: 144871-bp) from *Escherichia coli* isolates obtained from patients with urinary tract infections and one plasmid (pEC_Bactec: 92970-bp) from an *Escherichia coli* strain isolated from the joint of a horse with arthritis were determined. Plasmid pEC_Bactec belongs to the IncI1 group and carries two resistance genes: *bla*_TEM-1_ and *bla*_CTX-M-15_. It shares more than 90% homology with a previously published *bla*_CTX-M_-plasmid from *E. coli* of human origin. Plasmid pEC_B24 belongs to the IncFII group whereas plasmids pEC_L8 and pEC_L46 represent a fusion of two replicons of type FII and FIA. On the pEC_B24 backbone, two resistance genes, *bla*_TEM-1_ and *bla*_CTX-M-15_, were found. Six resistance genes, *bla*_TEM-1_, *bla*_CTX-M-15_, *bla*_OXA-1_, *aac6'-lb-cr*, *tetA* and *catB4*, were detected on the pEC_L8 backbone. The same antimicrobial drug resistance genes, with the exception of *tetA*, were also identified on the pEC_L46 backbone. Genome analysis of all 4 plasmids studied provides evidence of a seemingly frequent transposition event of the *bla*_CTX-M-15_-IS*Ecp1* element. This element seems to have a preferred insertion site at the *tnpA* gene of a *bla*_TEM_-carrying Tn*3*-like transposon, the latter itself being inserted by a transposition event. The IS*26*-composite transposon, which contains the *bla*_OXA-1_, *aac6'-lb-cr* and *catB4* genes, was inserted into plasmids pEC_L8 and pEC_L46 by homologous recombination rather than a transposition event. Results obtained for pEC_L46 indicated that IS*26* also plays an important role in structural rearrangements of the plasmid backbone and seems to facilitate the mobilisation of fragments from other plasmids.

**Conclusions:**

Collectively, these data suggests that IS*26* together with IS*Ecp1* could play a critical role in the evolution of diverse multiresistant plasmids found in clinical *Enterobacteriaceae*.

## Introduction

Most antibiotics used are naturally occurring chemical compounds produced by environmental fungi and bacteria, but also synthetic antibiotics (chemotherapeutic agents) are in circulation. In the course of evolution, bacteria have developed several strategies to deal with severe effects caused by these antimicrobial drugs. Bacterial plasmids play an important role in the horizontal transfer of antimicrobial drug resistance genes. The capability of plasmids to spread between bacterial cells by conjugation greatly enhances the dissemination of these resistance genes and raises a series of clinical problems [1]–[4].

β-lactam antibiotics are one, if not the most important group of antimicrobial agents in human and veterinary medicine. The predominant cause of resistance to β-lactams in Gram-negative bacteria is the production of β-lactamases. Both chromosome- and plasmid-encoded β-lactamases have been described. Until now, more than 400 β-lactamases have been reported and new β-lactamases continue to emerge worldwide [5]. Genes encoding extended-spectrum β-lactamases (ESBL), providing resistance to both penicillins and broad-spectrum cephalosporins but not carbapenems and β-lactamase inhibitors, are often located on conjugative plasmids [6], [7]. Plasmid-mediated ESBLs are most commonly of the TEM-, SHV- or CTX-M-type. CTX-M enzymes have become the most prevalent family of ESBLs among *Enterobacteriaceae* since their first report in 1986. To date, more than 80 CTX-M enzymes have been isolated. They are divided into 5 clusters on the basis of the amino acid sequence: CTX-M-1, CTX-M-2, CTX-M-8, CTX-M-9 and CTX-M-25 [8], [9].

Several studies have reported CTX-M-producing *Escherichia coli* strains as major global human pathogens, primarily associated with urinary tract infections. Notably, clinical CTX-M-15-producing *E. coli* isolates have become more and more widespread [7], [10]–[13]. Some plasmids, isolated from bacteria of human origin and carrying *bla*_CTX-M_ genes, have been studied in order to better understand their dissemination mechanisms [14]–[17]. Information on *bla*_CTX-M_-carrying plasmids from clinical isolates of animal origin is, however, lacking. Therefore, we determined the complete nucleotide sequence of a *bla*_CTX-M-15_-carrying plasmid originating from an *E. coli* isolated from a horse and compared this plasmid with a known plasmid from an *E. coli* isolate from human origin. This study also highlights the evolution of IncF plasmids by determining the complete nucleotide sequence of three CTX-M-15-encoding plasmids from *E. coli* isolates from humans, thereby enhancing our understanding of the pedigree of these plasmids.

## Results and Discussion

### Analysis of pEC_Bactec

**Figure 1:**
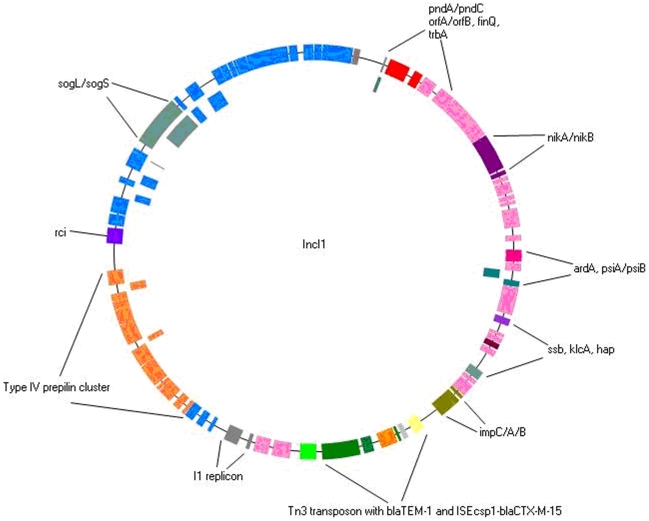
Physical map of pEC_Bactec (IncI1). The hypothetical proteins and predicted ORFs are presented by coloured boxes (blue for the *tra* locus, orange for the type IV prepilin cluster and pink for hypothetical proteins). The I1 replicon is coloured in grey. The *tnpA* genes are indicated by red boxes whereas the IS*26* element is bright green. Other genes that have an important function for pEC_Bactec bear different coloured boxes: green-brown, *impC*/*A*/*B* genes; blue-grey, *sogL*/*sogS* genes; blue-green, *psiA*/*psiB* genes; dark pink, *ardA* gene: purple, *nikA*/*nikB* genes; blue-purple, *rci* gene; red-brown, *klcA* gene; and dark-grey, *pndA*/*pndC*. The new Tn*3* element, containing *tnpA* (green) and *tnpR* (grey) genes and encoding CTX-M-15 (green) in addition to TEM-1 (yellow) is also highlighted. The IS*Ecp1* element linked to *bla*_CTX-M-15_ is coloured in orange.

Plasmid pEC_Bactec is a circular molecule of 92970-bp harbouring 86 open reading frames (ORFs) (Table S1). Conjugation experiments showed that it is transferable. pEC_Bactec belongs to the incompatibility group IncI1. pMLST assigned it to a new IncI1 pMLST type, sequence type (ST) 33 (*repI*3, *pilL*3, *sogS*3, *ardA*4, *trbA-pndC*15), which belongs to the clonal complex ST31 (http://pubmlst.org/perl/mlstdbnet/). The *trbA-pndC* region, one of the five selected alleles for pMLST, can vary in length due to the insertion of the *finQ* gene, encoding the fertility inhibitor, in the 5′ end of the *pndC* gene [18]. In addition to this *finQ* insertion, the pEC_Bactec plasmid contains an extra insertion of two IS*66* ORFs between *finQ* and *pndC* resulting in a longer *trbA-pndC* region (4939-bp) and a new allele variant *trbA-pndC*15 (Fig. 1). Similar IncI1 plasmids have been isolated from commensal *Enterobacteriaceae* of poultry and pigs [19]. However, these plasmids were of a different pMLST type, ST34 (*repI*3, *pilL*3, *sogS*3, *ardA*4, *trbA-pndC*16), although they belong to the same ST31-complex (unpublished data). It should be noted that for these plasmids the *trbA-pndC* region lacked the extra insertion of two IS*66* ORFs between *finQ* and *pndC*. All these findings may indicate that these plasmids, belonging to ST33 and ST34, are closely related and their prevalence is particularly to be linked to animal reservoirs.

The transfer region of R64, which is the reference plasmid for the IncI1 group, encoded the following genes: *traABCD*, *pilI-V*, *rci*, *traE-K*, *nuc*, *sogL*, *traL-Y*, *ecx*, *pnd*, *trbABC* and *nikBA* [20]. In comparison with R64, the transfer region of pEC_bactec is well conserved, with the exception of *traD* and *pilJ*, which appeared to be lost, and the *nuc* gene apparently rearranged within the transfer region. The *traD* and *pilJ* genes may not be required for plasmid transfer since pEC_Bactec was able to transfer in vitro by conjugation. In addition, the cluster encoding the type IV pili (PilI-V) is considered as a virulence factor. The association with resistance determinants may favour the dissemination of plasmids belonging to this plasmid family [21].

In comparison with R64 [20], pEC_Bactec lacks the arsenic, tetracycline and streptomycine resistance genes, the addiction systems *mck*/*kor* and *parA*/*parB*. This deleted region is replaced by its own resistance region (see below). The absence of these addiction systems suggests that they are not important in the maintenance of this plasmid.

**Figure 2:**
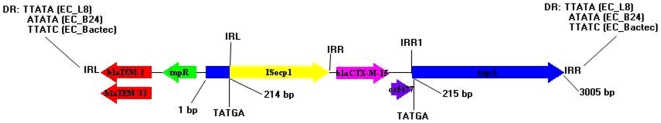
Detail of the new Tn*3* element, containing *tnpA* (disrupted, blue), *tnpR* (green), orf477 (purple), *bla*_CTX-M-15_ (pink), IS*Ecp1* (yellow) and *bla*_TEM-1_ or *bla*_TEM-33_ (red) genes. The 5-bp direct repeats consistent with transposition events of the Tn*3* transposon and the IS*Ecp1*- *bla*_CTX-M-15_ element are also shown, as are tags for inverted repeats (IR prefix) and basepair numbering corresponding to transposon-seperated segments of the *tnpA* gene.

Plasmid pEC_bactec carries only two known resistance genes, *bla*_TEM-1_ and *bla*_CTX-M-15_ (Fig. 1). The TEM-1 enzyme is only able to hydrolyse amino- and carboxy-penicillins. Previous studies have shown that *bla*_TEM_ genes are carried by three of the earliest described bacterial transposons, namely Tn*1*, Tn*2* and Tn*3*. These transposons contain the transposase and resolvase genes, *tnpA* and *tnpR*, as well as a *res* resolution site [22], [23]. The *bla*_TEM-1_ gene of pEC_Bactec was found to be located in a Tn*3*-like transposon possessing intact 38 bp inverted repeats (IRs) (Fig. 2). The Tn*3*-like transposon is flanked by 5-bp direct TTATC repeats functioning as a target site sequence. This indicates that this transposon has jumped from another plasmid or chromosome into this plasmid, a process called transposition. Interestingly, the *tnpA* gene of the Tn*3* transposon is disrupted by IS*Ecp1*-*bla*_CTX-M-15_ due to IS*Ecp1*-mediated transposition (Fig. 2). IS*Ecp1*-like elements belong to the IS*1380* family of insertion sequences and have been identified in association with genes belonging to the *bla*_CTX-M-1_, *bla*_CTX-M-2_, *bla*_CTX-M-25_ and *bla*_CTX-M-9_ ESBL gene clusters [24]–[27].

IS*Ecp1*-mediated transposition was shown to create a 5-bp duplication of the target sequence TATGA (Fig. 2). The mechanism involves the left inverted repeat (IRL) of IS*Ecp1* and a right inverted repeat (IRR1), which resembles the IRR of IS*Ecp1* (Fig. 2). Despite this disruption, the Tn*3 tnpA* gene still encodes a protein of 929 amino acids. If this truncated *tnpA* gene remains functional, it would mediate a new Tn*3* element encoding CTX-M-15 in addition to TEM-1. Based on these findings, we can conclude that pEC_Bactec arose by transposition of Tn*3* and IS*Ecp1*-*bla*_CTX-M-15_.

A similar disruption of the Tn*3*-like transposon by an IS*Ecp1*-*bla*_CTX-M-3_ element was recently reported for the IncI1 plasmid pEK204 found in a clinical *E. coli* isolate of human origin [16]. However, this IS*Ecp1*-*bla*_CTX-M-3_ element was inserted in an inverse way, which indicates that *bla*_CTX-M-3_ and *bla*_CTX-M-15_ represent separate escape events. Blast analysis showed 92% homology between pEC_Bactec and pEK204.

To our knowledge, this is the first genomic analysis of a *bla*_CTX-M-15_-carrying plasmid found in a clinical *E. coli* isolate from a horse. In different members of *Enterobacteriaceae* of human and animal origin, IncI1 plasmids encoding CTX-M-15 have been reported earlier [19], [21], [28]. On some IncI1 plasmids, carrying *bla*_CTX-M-15_, extra non-β-lactam resistance determinants were observed [28]. All these findings demonstrate the high plasticity of IncI1 plasmids.

### Analysis of pEC_B24

**Figure 3:**
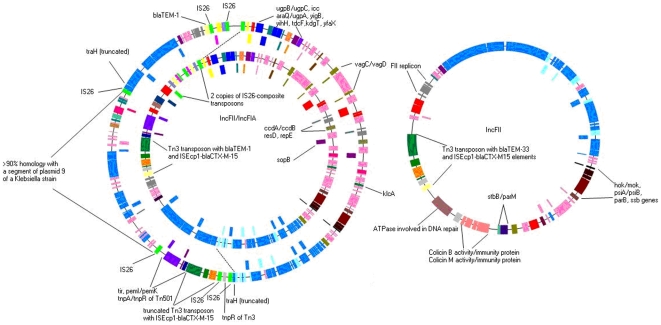
Physical maps of pEC_B24 (IncFII), pEC_L8 (IncFII, FIA) and pEC_L46 (IncFII, FIA). The hypothetical proteins and predicted ORFs are represented by coloured boxes. The pEC_L8 (inner circle) is compared with pEC_L46 (outer circle). Dashed lines stake out a large common region (right half). The *tra* locus is indicated by blue boxes and the hypothetical proteins are indicated in pink. The *tnpA* genes are indicated in red boxes whereas the IS*26* element is coloured in bright green. The antitoxin/toxin genes are indicated by green-brown (*vagC*/*vagD*), purple (*pemI*/*pemK*), blue-grey (*ccdA*/*ccdB*) and brown-black-red (*hok*/*mok*, *parB*, *psiA*/*psiB*), respectively. The FII replicon and FIA replicon is indicated in grey and grey-green, respectively. Other genes that have an important function for these IncF plasmids are indicated by different coloured boxes: dark blue (*ugpB*, *ugpC*, *araQ* and *ugpA*), dark green (*icc*, *klcA*), green (*yigB*), purple (*sopB*, *kdgT*), orange (*yihH*, *tdcF*), orange-pink (colicin B/M genes) and purple blue (*yfaX*). The new Tn*3* element, containing *tnpA* (green) and *tnpR* (grey) genes and encoding CTX-M-15 (green) in addition to TEM-1 (yellow) is also highlighted. The IS*Ecp1* element linked to *bla*_CTX-M-15_ is coloured orange. The IS*26* (bright-green) composite transposon carrying *aac6'-lb-cr* (purple-blue), *bla*_OXA-1_ (light yellow) and *catB4* (pink) is also shown as is the pEC_L46 fragment with more than 90% homology to a segment from a *Klebsiella* plasmid.

Plasmid pEC_B24 is a circular molecule of 73801-bp harbouring 76 open reading frames (ORFs) (Table S2, Fig. 3). This plasmid was transferred in vitro by conjugation and belongs to the incompatibility group IncFII.

Comparative analysis with another IncF plasmid (pC15a-1a) [14] revealed that both pEC_B24 and pC15a-1a plasmids carry the FII replicon (*repA2*, *repA3*, *repA1* and *repA4*), *hok*/*mok*, *psiA*/*psiB* and *parB* genes and the type I partitioning locus (*parM*, *stbB*) ensuring stable plasmid inheritance. The transfer region of pEC_B24 comprises 19 *tra* genes (*traE*, *traK*, *traB*, *traP*, *traV*, *traR*, *traC*, *traW*, *traU*, *traN*, *traF*, *traQ*, *traH*, *traG*, *traS*, *traT*, *traD*, *traI* and *traX*), 7 *trb* genes (*trbG*, *trbI*, *trbC*, *trbE*, *trbA*, *trbB* and *trbJ*) and *finO*. In comparison with the transfer region of other IncFII plasmids [29], [30], several *tra* genes (*traM*, *traJ*, *traY*, *traA* and *traL*), the *trbF* and *artA* genes are missing. Despite this incompleteness, pEC_B24 is easily transferred by conjugation.

Another interesting finding on this plasmid is the presence of colicin B and M genes. These transmembrane toxins kill sensitive strains of *E. coli* and closely related species by depolarising the cytoplasmic membrane, which lead to dissipation of cellular energy [31]. To our knowledge, colicin B and M genes have never been described on IncFII plasmids. Whether these genes have an influence on the spread of this plasmid remains unclear and needs further investigation.

Genes encoding antimicrobial resistance are *bla*_TEM-33_ and *bla*_CTX-M-15_ (Fig. 3). TEM-33, also called inhibitor resistant TEM 5 or IRT-5, has a reduced affinity for β-lactamase inhibitors. IRT enzymes have negligible activity against extended-spectrum cephalosporins and are not considered to be ESBLs [32]. The *bla*_TEM-33_ gene of pEC_B24 is located in a Tn*3*-like transposon possessing intact 38-bp inverted repeats (IRs). Like in pEC_Bactec, the Tn*3*-like transposon was inserted after a transposition event and is flanked by 5-bp directed repeats (ATATA) (Fig. 2). Likewise, the Tn*3 tnpA* gene is disrupted by IS*Ecp1*-*bla*_CTX-M-15_ due to IS*Ecp1*-mediated transposition creating a 5-bp duplication of the target sequence TATGA (Fig. 2). The IS*Ecp1*-*bla*_CTX-M-15_ element seems to show high affinity for this target sequence because its transposition was also described for another IncFII plasmid, pC15a-1a [14]. BLAST analysis yielded 72, 44 and 41% homology between pEC_B24 and previously described IncFII plasmids pC15a-1a, pEK499 and pEK516, respectively. Thus, pEC_B24 represents a new variant of IncFII plasmids harbouring *bla*_CTX-M-15_.

### Analysis of pEC_L8 and pEC_L46

Plasmids pEC_L8 and pEC_L46 are both circular molecules counting 118525 and 144871-bp with 133 and 162 ORFs, respectively. Both plasmids were transferred by conjugation, belong to the incompatibility group F and represent two replicons of type FII and FIA (Table S3 and S4, Fig. 3). Comparative analysis revealed 80% homology between both plasmids.

Both plasmids also comprise several addiction systems to ensure stable plasmid inheritance and post-segregation killing: (1) the post-segregation killing protein Hok and its modulator Mok, both located near the *parB* and *psiA*/*psiB* genes; (2) the toxin-antitoxin system *pemI*/*pemK*; (3) two copies of the *vagC*/*vagD* virulence-associated genes; (4) and the toxin-antitoxin system *ccdA*/*ccdB*, located near the FIA replicon (Fig. 3). These addiction systems have been described in other IncF plasmids [16], [29], [30]. They may explain the success in the maintenance of these plasmids even in the absence of antibiotic selective pressure.

Both plasmids also share a region with two copies of genes encoding permeases and ATP-binding proteins of the ABC transporter family. Whether these transporters contribute to virulence and plasmid maintenance remains unknown and needs further investigation.

The pEC_L8 and pEC_L46 plasmids had a complete transfer region: 24 *tra* genes (*traM*, *traJ*, *traY*, *traA*, *traL, traE*, *traK*, *traB*, *traP*, *traV*, *traR*, *traC*, *traW*, *traU*, *traN*, *traF*, *traQ*, *traH*, *traG*, *traS*, *traT*, *traD*, *traI* and *traX*), 8 *trb* genes (*trbG*, *trbI*, *trbC*, *trbE*, *trbA*, *trbB*, *trbJ* and *trbF*) and *finO*. In comparison with other IncF plasmids [29], [30], these plasmids acquired an extra *trbD* and *traR* gene. This complete *tra* region makes them self-transmissible.

**Figure 4:**
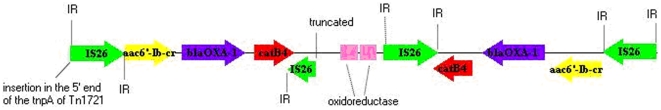
Detail of two IS*26*-composite transposon elements carrying the *bla*_OXA-1_, *aac6'-lb-cr* and *catB4* genes, which are inserted in an inverted orientation. Both elements are separated by two oxidoreductase (*yigB* genes) elements. The intact left and right inverted repeats of IS*26* are also shown

Plasmid pEC_L8 carries six genes encoding antimicrobial resistance: *bla*_TEM-1_, *bla*_CTX-M-15_, *bla*_OXA-1_ (oxacillin-hydrolizing capabilities, not regarded as ESBLs), *aac6'-lb-cr* (encodes an aminoglycoside acetyltransferase that also modifies ciprofloxacin and norfloxacin), *tetA* (encoding resistance to tetracyclines) and *catB4* (chloramphenicol resistance). All these resistance genes, with the exception of the *tetA* gene, were also found on the pEC_L46 plasmid backbone. The *tetA* gene is located in a Tn*1721*-like transposon. This mobile genetic element has been described previously on plasmid backbones of the IncA/C group [33] which indicates that its appearance is not limited to plasmids of the IncF group. The resistance genes *bla*_OXA-1_, *aac6'-lb-cr* and *catB4* are found in an IS*26*-composite transposon of which the IS*26* elements lie in opposite orientations (Fig. 4). Examination of the pEC_L8 backbone revealed two IS*26*-composite transposon elements, which were inserted in an inverted orientation. Both transposons are separated by two oxidoreductase (*yigB* genes) elements (Fig. 4). Interestingly, the IS*26* element of one composite transposon element, located downstream of the two *yigB* genes, is truncated (left composite transposon in Fig. 4). To our knowledge, this is the first observation of this type of insertional arrangement. However, it is unclear whether this duplication provides any advantage in terms of resistance to oxacillin, aminoglycosides and chloramphenicol and needs further investigation. Furthermore, the 5′end of the *aac6'-lb-cr* gene truncates one IS26 element, and the 3′end of the *catB4* gene is truncated by the other IS*26* element (Fig. 4). Extensive analysis of IS*26* revealed promoter-like elements in which the putative -35 box (TTGCAA) was found in the left inverted repeat of IS*26* and the putative -10 box was found 14-bp upstream of the −35 box. This may provide a mechanism for expression of the downstream resistance genes. A similar hybrid promoter involving IS*26*-inverted repeat sequences was previously shown to steer the expression of aminoglycoside resistance genes located in a composite transposon [34].

The *bla*_TEM-1_ gene of pEC_L8 is contained in a Tn*3*-like transposon possessing intact 38-bp inverted repeats (IRs) (Fig. 2). Inserted after a transposition event, the transposon is flanked by 5-bp directed repeats of TTATA. Likewise, the *tnpA* gene is disrupted by IS*Ecp1*-*bla*_CTX-M-15_ due to IS*Ecp1*-mediated transposition creating a 5-bp duplication of the target sequence TATGA (Fig. 2). The latter transpositional event also occurred in pEC_L46. However, the new Tn*3* transposon was rearranged by IS*26* elements so that the *tnpR* gene is now flanked by IS*26*. The *bla*_TEM-1_ gene is situated further downstream on the pEC_L46 backbone (Table S4, Fig. 3) and its 5′end is disrupted by an IS*26* element.

Further examination of the pEC_L46 backbone revealed more structural rearrangements. Interestingly, all these rearrangements were also flanked by IS*26* elements with intact left and right inverted repeats (Fig. 3). This shows the importance of IS*26* in the plasticity of plasmids.

A major difference between pEC_L8 and pEC_L46 is the insertion of a 29.5-kb fragment flanked by IS*26* in the pEC_L46 backbone (Table S4, Fig. 3). This fragment showed more than 90% homology with a segment of the previously described plasmid S9 from a *Klebsiella* strain [35]. This finding indicates that genetic exchange between S9 and pEC_L46 may have happened and that the insertion sequence IS*26* played a crucial role in this process. Insertion of this large fragment provided pEC_L46 with extra hypothetical proteins, *tra* genes (two copies for some) and genes encoding antirestriction proteins (Table S4).

The antirestriction proteins, ArdK and ArdR, may play a role in overcoming the host restriction barrier by self-transmissible broad-host range plasmids. They can serve as a genetic switch that controls the expression of plasmid-encoded antirestriction functions during mating [36]. Whether the extra *tra* genes could affect the transfer of this plasmid by conjugation remains unclear and needs further investigation.

None of the IS*26* elements, responsible for the structural rearrangements of the pEC_L46 backbone or flanking the 29.5-kb fragment, and those flanking the composite transposons of both pEC_L8 and pEC_L46, showed target site duplications. Moreover, the absence of a direct target sequence duplication at either side of these IS*26*-flanked regions strengthens the hypothesis of entry through homologous recombination rather than transposition.

Nevertheless, transpositional events creating direct target sequence duplications have been discovered for certain IS*26*-composite transposons [37], [38]. In view of the wide spread of IS*26* among plasmids, the acquisition of resistance genes and other genetic fragments might be realized in different ways.

Blast analysis of pEC_L8 and pEC_L46 against pEK499, another IncF plasmid encoding *bla*_CTX-M-15_, resulted in 79% and 75% homology, respectively. Plasmids of the IncF group are geographically widespread and have played a crucial role in the spread of CTX-M-15 in *Enterobacteriaceae* among humans [16].

### Concluding remarks

We have determined the complete nucleotide sequence of four CTX-M-15-encoding plasmids carried by three clonally distinct clinical *E. coli* isolates of human origin and one clinical *E. coli* isolate from a horse. For the first time, the nucleotide sequence of a *bla*_CTX-M15_-carrying plasmid (IncI1) of a clinical isolate from animal origin is described. Comparative analysis of this plasmid with a *bla*_CTX-M-3_-carrying plasmid (IncI1) of a clinical isolate of human origin [16] revealed a high degree of homology (>90%), indicating that similar plasmids carrying different *bla*_CTX-M_ genes are circulating both in the human and animal ecosystem. Notably, more plasmid genomic research is necessary to ascertain whether the human and animal environment could be experienced by bacteria in a different way.

This study also highlights the evolution of IncF plasmids, thereby enhancing our understanding of the pedigree of these plasmids carrying *bla*_CTX-M_ genes. The multitude of addiction systems present on these IncF plasmids ensures their maintenance even in the absence of antibiotic selection.

This report has also provided support for the seemingly frequent transposition events of the *bla*_CTX-M-15_ gene linked to IS*Ecp1*. This element seems to have a preferred insertion site at the *tnpA* gene of a *bla*_TEM_-carrying Tn*3*-like transposon, the latter also being inserted by a transposition event. Both transposition events prefer AT-rich target sequences, whereas the IS*Ecp1*-*bla*_CTX-M-15_ element prefers the same target sequence for all Tn*3* elements. Whether this new Tn*3* element, encoding *bla*_CTX-M-15_ in addition to *bla*_TEM_, remains mobile is still unclear. The potential for transposition of two *bla* genes, of which one is an ESBL gene, has public health implications since extended-spectrum cephalosporins are extensively used in human and veterinary medicine. This needs further investigation.

The mobile genetic element, IS*26*, member of the IS*6* family, is shown to play an important role in the plasticity of the investigated plasmids. It inserts by homologous recombination, as indicated by the absence of target site duplication, and causes rearrangements. IS*26* seems not only to facilitate the mobilisation of chromosomal fragments [39] but also fragments from other plasmids. Collectively, these data suggest that IS*26* and IS*Ecp1* play a critical role in the evolution of diverse multiresistant plasmids found in clinical *Enterobacteriaceae*.

## Materials and Methods

### Bacterial isolates

*E. coli* B24, L8 and L46 were isolated in Belgium from human patients with urinary tract infections. B24 was collected from a urine sample from a hospitalized patient of the Ghent University Hospital. L8 and L46 were defined as community-acquired isolates from urine samples obtained from a medical centre serving only general practitioners in Leuven. All isolates belonged to sequence type ST131 and showed clonally distinct PFGE-fingerprint patterns [40]. *E. coli* Bactec was isolated from the joint of a horse suffering arthritis at the Faculty of Veterinary Medicine, Ghent University. All isolates were shown to produce CTX-M-15 by PCR and sequencing [41]. All isolates were available at the start of this study and were gathered as part of standard care.

Approval of the Ethics Review Board at the University of Ghent was not necessary, because no information about the patients was released and the identified *E. coli* isolates were exempt from requirements for ethical approval.

### Isolation of plasmids

Plasmid transfer experiments were carried out in Luria Broth medium. *E. coli* J5, resistant to rifampicin, was used as recipient strain. Conjugation experiments were performed overnight at 37°C with a donor/recipient ratio of 0.2. Transconjugants were selected on MacConkey agar plates (Oxoid LTD, Basingstoke, Hampshire, England) supplemented with ceftiofur (8 mg/liter) and rifampicin (250 mg/liter). Plasmid DNA was isolated from the transconjugants using a plasmid midi Qiagen kit (Venlo, the Netherlands) according to the manufacturer's instructions and separated on 0.8% 1x TAE agarose gels by electrophoresis at 140V for 4 h at 4°C. The molecular size of each ESBL-carrying plasmid was estimated by using a BAC Tracker Supercoiled DNA ladder (ranging from 165 kb to 8 kb) (Epicentre Biotechnologies, Madison, Wisconsin).

### Plasmid sequencing and sequence assembly

Twenty µg of each plasmid DNA was extracted and purified as described above. Roche GS-FLX titanium libraries were generated, using 5 µg of each of the 4 purified plasmid DNA samples. The DNA was fragmented by nebulisation, followed by a double Solid Phase Reversible Immobilization (SPRI) bead capture size selection with Ampure beads (Agencourt Bioscience) to generate DNA fragments of 400–1,500 bp in length. Selected fragments were end-repaired and ligated to 454 sequencing adapters. Single-stranded libraries were then generated according to the Roche GS FLX Titanium General Library Preparation Method Manual (version October 2008). These single-stranded libraries were used to perform an emulsion PCR according to the Roche GS FLX titanium emPCR Method Manual (version October 2008). The 4 resulting bead libraries were sequenced on a Roche GS-FLX system following the GS FLX Titanium Sequencing Method Manual (version October 2008). A 70×75 mm picotiter plate was divided in 8 lanes using a rubber gasket. For each of the 4 bead libraries, 1 lane was loaded with 340.000 DNA library beads.

The Mimicking Intelligent Read Assembly package MIRA (version 2.9.43) was used to perform *de novo* genome assembly. For the pEC_Bactec sample, 50891 of the 55632 generated sequences were assembled into one relevant contig of 93799-bp. The start and the end of this contig showed significant overlap and represented the complete, circular sequence of the pEC_Bactec plasmid. The average sequencing coverage for this plasmid was 179x. For the pEC_B24 sample, 23915 of the 25319 generated sequences were assembled into one relevant contig of 74322-bp. The start and the end of the 74322-bp contig showed significant overlap and represented the complete, circular sequence of the pEC_B24 plasmid. The average sequencing coverage for this plasmid was 112x. For the pEC_L8 sample, 54048 of the 58671 generated sequences were assembled into five relevant contigs ranging from 64755 to 7430 bp. Walking reads were used to assemble the contigs and to fill-in the gaps. The average sequencing coverage for this plasmid was 109x. For the pEC_L46 sample, 31381 of the 35029 generated sequences were assembled into 4 relevant contigs ranging from 65414 to 5267 bp. Walking reads were used to assemble the contigs and to fill-in the gaps. The average sequencing coverage for this plasmid was 71x.

### Bioinformatics analysis (annotation)

In order to maximize the number of quality gene annotations, an *ab initio* annotating approach was followed.

Theoretical open reading frames (ORFs) were first determined using the EMBOSS getorf tool (with minimum ORF length set to 90 nucleotides, and taking all alternative start codons into account). All ORFs were translated subsequently, and BLAST (more precisely the blastp program of the BLAST suite) [42] was performed with an e threshold of 10^−15^ against the Uniprot/KB universal protein database. The generalist algorithm of getorf yielded roughly a tenfold of the expected natural ORFs, reducing the risk of false negatives. In order to keep the false positive rate low, extra parameters were considered: 1) percentage alignment between query and hit ORFs; 2) percentage conservation between aligned portions of query and hit ORFs; 3) ribosome binding strength (see details further down). ORFs with a bad ranking value (arbitrary but consistent cutoff for all comparisons) for any two of these three parameters were considered as false positives and discarded.

Ribosome binding strength was estimated by applying two long established facts.

(1) On an mRNA strand, usually within 20 nucleotides before the actual start codon, the reverse complement of 5 to 7 nucleotides near the 16S rRNA 3′ end acts as an attractor and positioner for the ribosomal small subunit (which contains the 16S rRNA in addition to a set of ribosomal proteins). This sequence appears to be more than averagely conserved and is known as the Shine-Dalgarno sequence [43], [44]; (2) In Gram-negative bacteria such as organisms belonging to the former *Proteobacteria* division (which includes *Escherichia*), an AU-rich mRNA region of 16 nucleotides long and immediately preceding the Shine-Dalgarno sequence may also attract and position ribosomes to help initiate translation of the correct, biologically active gene product [45], [46]. For *E. coli*, the Shine-Dalgarno sequence was determined to be a subsequence of AGGAGGU (which is the reverse complement of the 3′ end of the 16S rRNA), and the minimum AU-richness (equivalent to ribosome binding capacity) of the preceding region was arbitrarily set to 10/16. For each theoretical ORF a range of possible start codons was scored; the higher the similarity to the ideal Shine-Dalgarno sequence, or the AU-richer the preceding region, or the better a combination of both, the more likely the potential start codon is to be the actual start codon.

For each BLAST hit, the following annotation information was either parsed from the BLAST report or further derived from parsed data (all in an automated fashion via Perl and Bioperl scripting): 1) the full query (*E. coli*) plasmid-derived ORF with coordinates (contig, theoretical ORF number, frame, ORF length, start and stop position); 2) corresponding hit parameters if relevant for further comparison; 3) remarks about ORF shortcomings (e.g. missing or extra domains with regard to the reference ORF, ORF interruption upon hitting a contig end) encountered during the parsing and annotation process; 4) an estimate of the ribosome binding strength of the mRNA region preceding the start codon; 5) nucleic acid sequence of the ORF; 6) BLAST alignment data (e value, number and fraction of amino acids unaligned, fraction of identical and fraction of conserved amino acids); 7) gene name and description if available.

Sequences of the open reading frames were compared and aligned with Genbank data using BLAST and with reference plasmids (R64 (NC_005014.1; reference plasmid for the IncI1 group), pEK204 (EU935740), pEK499 (EU935739), pEK516 (EU935738) and pC15a-1a (AY458016) by two sequence alignment using Blast2seq (http://blast.ncbi.nih.gov/Blast.cgi).

### Plasmid multilocus sequence typing (pMLST)

The pMLST scheme for IncI1 plasmids was used for EC_Bactec as described previously [18]. The *pilL* (254 bp), *sogS* (254 bp), *ardA* (343 bp), *repI1* (104 bp) and *tnbA-pndC* (812 bp) fragments were compared with known allelic variants (http://pubmlst.org/perl/mlstdbnet/mlstdbnet.pl?file=incI1_profiles.xml&page=oneseq).

### Nucleotide sequences

pEC_Bactec (GU371927), pEC_B24 (GU371926), pEC_L8 (GU371928), pEC_L46 (GU371929).

## Supporting Information

Table S1 (0.12 MB DOC)

Table S2 (0.11 MB DOC)

Table S3 (0.17 MB DOC)

Table S4 (0.20 MB DOC)

## References

[1] Bywater RJ (2004). Veterinary use of antimicrobials and emergence of resistance in zoonotic and sentinel bacteria in the EU.. J Vet Med.

[2] Bywater RJ (2005). Identification and surveillance of antimicrobial resistance dissemination in animal production.. Poultry Sci.

[3] Davies J (1994). Inactivation of antibiotics and the dissemination of resistance genes.. Science.

[4] Frost LS, Leplae R, Summers AO, Toussaint A (2005). Mobile genetic elements: the agents of open source evolution.. Nat Rev Microbiol.

[5] Bradford PA (2005). β-lactamases in the 21^st^ century: characterization, epidemiology and detection of this important resistance threat.. Clin Microbiol Rev.

[6] Horish RE (2002). Ceftiofur use in food animals.. Curr Top Med Chem.

[7] Pitout JDD, Laupland KB (2008). Extended-spectrum β-lactamase-producing *Enterobacteriaceae*: an emerging public-health concern.. The Lancet.

[8] Gupta V (2007). An update on newer β-lactamases.. Indian J Med Res.

[9] Paterson DL, Bonomo RA (2005). Extended-spectrum β-lactamases: a clinical update.. Clin Microb Rev.

[10] Karim A, Poirel L, Nagarajan S, Nordmann P (2001). Plasmid-mediated extended-spectrum β-lactamase (CTX-M-3 like) from India and gene association with insertion sequence IS*Ecp1*.. FEMS Microbiol Letters.

[11] Mushtaq S, Woodford N, Potz N, Livermore DM (2003). Detection of CTX-M-15 extended-spectrum β-lactamase in the United Kingdom.. J Antimicrob Chemother.

[12] Ruppé E, Hem S, Lath S, Gautier V, Ariey F (2009). CTX-M β-lactamases in *Escherichia coli* from community-acquired urinary tract infections, Cambodia.. Emerg Infect Dis.

[13] Sidjabat HE, Paterson DL, Adams-Haduch JM, Ewan L, Pasculle AW (2009). Molecular epidemiology of CTX-M-producing *Escherichia coli* at a Tertiary medical center in Western Pennsylvania.. Antimicrob Agents Chemother.

[14] Boyd DA, Tyler S, Christianson S, McGeer A, Matthew PM (2004). Complete nucleotide sequence of a 92-kilobase plasmid harbouring the CTX-M-15 extended-spectrum beta-lactamase involved in an outbreak in long-term care facilities in Toronto, Canada.. Antimicrob Agents Chemother.

[15] Shen P, Jiang Y, Zhou Z, Zhang J, Yu Y, Li L (2008). Complete nucleotide sequence of pKP96, a 67 850 bp multiresistance plasmid encoding *qnrA1*, *aac(6')-lb-cr* and *bla*
_CTX-M-24_ from *Klebsiella pneumoniae*.. Antimicrob Agents Chemother.

[16] Woodford N, Carattoli A, Karisik E, Underwood A, Ellington MJ, Livermore DM (2009). Complete nucleotide sequence of plasmids pEK204, pEK499 and pEK516, encoding CTX-M enzymes in three major *Escherichia coli* lineages from the United Kingdom, all belonging to the international O25:H4-ST131 clone.. Antimicrob Agents Chemother.

[17] Zhu WH, Luo L, Wang JJ, Zhuang XH, Zhong L (2009). Complete nucleotide sequence of pCTX-M360, an intermediate plasmid between pEL60 and pCTX-M-3, from a multidrug resistant *Klebsiella pneumoniae* strain isolated in China.. Antimicrob Agents Chemother.

[18] Garcia-Fernandez A, Chiaretto G, Bertini A, Villa L, Fortini D (2008). Multilocus sequence typing of IncI1 plamsids carrying extended-spectrum beta-lactamases in Escherichia coli and Salmonella of human and animal origin.. J Antimicrob Chemother.

[19] Smet A, Martel A, Persoons D, Dewulf J, Heyndrickx M (2009). Comparitive analysis of Extended-spectrum-β-lactamase (ESBL)-carrying plasmids from different members of *Enterobacteriaceae* isolated from poultry, pigs and humans: evidence for a shared β-lactam resistance gene pool?. J Antimicrob Chemother.

[20] Komano T, Yoshida T, Narahara K, Furuya N (2000). The transfer region of IncI1 plasmid R64: similarities between R64 *tra* and *Legionella icm/dot* genes.. Mol Microb.

[21] Carattoli A (2009). Resistance plasmid families in *Enterobacteriaceae*.. Antimicrob Agents Chemother.

[22] Heffron F, McCarthy BJ, Ohtsubo E (1979). DNA sequence analysis of the transposon Tn*3*: three genes and three sites involved in transposition of Tn*3*.. *Cell*.

[23] Partridge SR, Hall RM (2005). Evolution of transposons containing *bla*
_TEM_ genes.. Antimicrob Agents Chemother.

[24] Bae IK, Lee BH, Hwang HY, Jeong SH, Hong SG (2006a). A novel ceftazidime-hydrolising extended-spectrum β-lactamase, CTX-M-54, with a single amino acid substitution at position 167 in the omega loop.. J Antimicrob Chemother.

[25] Bae IK, Lee YN, Hwang HY, Jeong HS, Lee SJ (2006b). Emergence of CTX-M-12 extended-spectrum β-lactamase-producing *Escherichia coli* in Korea.. J Antimicrob Chemother.

[26] Eeckert C, Gautier V, Arlet G (2006). DNA sequence analysis of the genetic environment of various *bla*
_CTX-M_ genes.. J Antimicrob Chemother.

[27] Fernandez AG, Cloeckaert A, Bertini A, Praud K, Doublet B (2007). Comparative analysis of Inc*HI2* plasmids carrying *bla*
_CTX-M-2_ or *bla*
_CTX-M-9_ from *Escherichia coli* and *Salmonella enterica* strains isolated from poultry and humans.. Antimicrob Agents Chemother.

[28] Hopkins KL, Liebana E, Villa L, Batchelor M, Threlfall EJ, Carattoli A (2006). Replicon typing of plasmids carrying CTX-M or CMY β-lactamases circulating among *Salmonella* and *Escherichia coli* isolates.. Antimicrob Agents Chemother.

[29] Johnson TJ, Siek KE, Johnson SJ, Nolan LK (2005). DNA sequence and comparative genomics of pAPEC-O2-R, an avian pathogenic Escherichia coli transmissible R plasmid.. Antimicrob Agents Chemother.

[30] Périchon B, Bogaerts P, Lambert T, Frangeul L, Courvalin P, Galimand M (2008). Sequence of conjugative plasmid pIP1206 mediating resistance to aminoglycosides by 16s rRNA methylation and to hydrophilic fluoroquinolones by efflux.. Antimicrob Agents Chemother.

[31] Schramm E, Mende J, Braun V, Kamp RM (1987). Nucleotide sequence of the colicin B activity gene cba: consensus pentapeptide among TonB-dependent colicins and receptors.. J Bacteriol.

[32] Chaibi EB, Sirot D, Paul G, Labia R (1999). Inhibitor resistant TEM β-lactamases: phenotypic genetic and biochemical characteristics.. J Antimicrob Chemother.

[33] Rhodes G, Huys G, Swings J, McGann P, Hiney M (2000). Distribution of oxytetracycline resistance plasmids between aeromonads in hospital and aquaculture environments: implication of Tn*1721* in dissemination of the tetracycline resistance determinant Tet A.. Appl Environ Microbiol.

[34] Lee KY, Hopkins JD, Syvanen M (1990). Direct involvement of IS*26* in an antibiotic resistance operon.. J Bacteriol.

[35] Gootz TD, Lescoe MK, Dib-Hajj F, Dougherty BA, He W (2009). Genetic organization of transposase regions surrounding *bla*
_KPC_ carbapenamase genes on plamsids from *Klebsiella* strains isolated in a New York City Hospital.. Antimicrob Agents Chemother.

[36] Belogurov AA, Delver EP, Rodzevich OV (1993). Plasmid pKM101 encodes two nonhomologous antirestriction proteins (ArdA and ArdB) whose expression is controlled by homologous regulatory sequences.. J Bacteriol.

[37] Iida S, Mollet B, Meyer J, Arber W (1984). Functional characterization of the prokaryote mobile genetic element IS*26*.. Mol Gen Genet.

[38] Naas T, Mikamin Y, Imai T, Poirel L, Nordmann P (2001). Characterization of In53, a class 1 plasmid-and composite transposon-located integron of *Escherichia coli* which carries an unusual array of gene cassettes.. J Bacteriol.

[39] Miriagou V, Carattoli A, Tzelepi E, Villa L, Tzouvelekis, LS (2005). IS*26*-associated In4-type integrons forming multiresistance loci in enterobacterial plasmids.. Antimicrob Agents Chemother.

[40] Smet A, Martel A, Persoons D, Dewulf J, Heyndrickx (2010). Characterization of extended-spectrum β-lactamases produced by *Escherichia coli* isolated from hospitalised and non-hospitalised patients: emergence of CTX-M-15-producing strains causing urinary tract infections.. Microb Drug Res in press.

[41] Smet A, Martel A, Persoons D, Dewulf J, Heyndrickx M (2008). Diversity of extended-spectrum β-lactamases and class C β-lactamases among cloacal *Escherichia coli* in Belgian broiler farms.. Antimicrob Agents Chemother.

[42] Altschul SF, Madden TL, Schäffer AA, Zhang J, Zhang Z (1997). Gapped BLAST and PSI-BLAST: a new generation of protein database search programs.. Nucleic Acids Res.

[43] Mikonnen M, Vuoristo J, Alatossava T (1994). Ribosome binding site consensus sequence of *Lactobacilus delbrueckii*.. FEMS Microbiol Lett.

[44] Shine J, Dalgarno L (1974). The 3′-terminal sequence of *E. coli* 16S ribosomal RNA: complementarity to nonsense triplets and ribosome binding sites.. Proc Natl Acad Sci USA.

[45] Boni IV, Isaeva DM, Musychenko ML, Tzareva NV (1991). Ribosome-messenger recognition: mRNA target sites for ribosomal protein S1.. Nucleic Acids Res.

[46] Komarova AV, Tchufistova LS, Dreyfus M, Boni IV (2005). AU-rich sequences within 5′ untranslated leaders enhance translation and stabilize mRNA in *Escherichia coli*.. J Bacteriol.

