# Supplementary material for: Complete Nucleotide Sequence of CTX-M-15-Plasmids from Clinical Escherichia coli Isolates: Insertional Events of Transposons and Insertion Sequences
Source: PLoS One. 2010 Jun 18;5(6):e11202. doi: 10.1371/journal.pone.0011202 (PMC2887853; doi:10.1371/journal.pone.0011202)
Supplement: Table S1 — (0.12 MB DOC) [file pone.0011202.s001.doc]

**Table S1.** ORFs identified in EC_Bactec (92970 bp).

| **Open reading frame (ORF)** | **Position (bp)** | **Protein function** |
| --- | --- | --- |
| *traX* | 1-585 | F pilin acetylation |
| *traY* | 613-2850 | Integral membrane protein |
| *ecx* | 2896-3366 | Surface exclusion protein |
| *pndA* | Compl. 5195-5345 | Post-segregation killing protein |
| *pndC* | Compl. 5197-5481 | Counter protein for PndA |
| *orfB* | Compl. 5575-7146 | IS66 tnpA |
| *orfA* | Compl. 7517-8193 | IS66 tnpA |
| *finQ* | 8364-9389 | Fertility inhibitor |
| *trbA* | 9762-10970 | Hypothetical protein |
| *trbB* | 10983-12050 | Hypothetical protein |
| *trbC* | 12046-14334 | Hypothetical protein |
| *nikB* | Compl. 14379-17078 | Relaxase |
| *nikA* | Compl. 17213-17544 | Relaxome component protein |
| *yggA* | 17649-17990 | Hypothetical protein |
| *ydiA* | Compl. 18076-18927 | Hypothetical protein |
| *ydhA* | Compl. 19059-19429 | Hypothetical protein |
| *ygeA* | 19504-19755 | Hypothetical protein |
| *ydgA* | Compl. 20043-20968 | Hypothetical protein |
| *ccgAII* | Compl. 20975-21438 | Hypothetical protein |
| *ydfA* | Compl. 21997-22430 | Hypothetical protein |
| *ardA* | Compl. 23076-23965 | Anti-restriction protein |
| *gp43* | Compl. 24050-24645 | Hypothetical protein |
| *psiA* | Compl. 24609-25328 | Plasmid SOS inhibition protein A |
| *psiB* | Compl. 25325-25762 | Plasmid SOS inhibition protein B |
| *yfhA* | Compl. 25803-27850 | Hypothetical protein |
| *ssb* | Compl. 28122-28649 | Single-stranded DNA-binding protein |
| *ychA* | Compl. 29289-29554 | Hypothetical protein |
| *orf73* | Compl. 29606-30028 | Hypothetical protein |
| *klcA* | Compl. 30079-30503 | Probable antirestriction protein |
| *yubH* | Compl. 30617-31092 | Hypothetical protein |
| *hap* | Compl. 32406-33089 | Hemaglutinin-associated protein |
| *O2R_79* | Compl. 33166-33510 | Hypothetical protein |
| *yccB* | Compl. 33595-34565 | Hypothetical protein |
| *impC* | 34612-34857 | UV protection and mutation protein |
| *impA* | 35010-35444 | UV protection and mutation protein |
| *impB* | 35423-36691 | UV protection and mutation protein |
| *bla*_TEM-1_ | Compl. 38154-39014 | Beta-lactamase TEM-1 precursor |
| *tnpR* | Compl. 39461-39856 | Tn3 resolvase |
| *tnpA* | 40018-40211 | Tn3 transposase (part 1) |
| IS*Ecp1* | 40315-41574 | Transposase |
| *bla*_CTX-M-15_ | 42095-42869 | Beta-lactamase CTX-M-15 precursor |
| *tnpA* | 43107-45893 | Tn3 transposase (part 2) |
| *tnpA* | 46308-47516 | Transposase of IS26 |
| *yagA* | Compl. 48209-49554 | Hypothetical protein |
| *yafB* | 49799-50401 | Hypothetical protein |
| *yafA* | 50418-50909 | Hypothetical protein |
| *repA4* | 51191-51466 | Regulatory protein |
| *repA* | Compl. 52085-53160 | Replication protein of the I1 replicon |
| *traA* | 54200-54484 | F pilin subunit |
| *traB* | 54908-55438 | F pilus assembly |
| *traC* | 55731-56411 | F pilus assembly |
| *pilI* | 56430-56726 | Type IV prepilin cluster |
| *pilK* | 56971-57564 | Type IV prepilin cluster |
| *pilL* | 57869-58933 | Type IV prepilin cluster, Lipoprotein |
| *pilM* | 58960-59249 | Type IV prepilin cluster |
| *pilN* | 59369-61048 | Type IV prepilin cluster, secretin protein |
| *pilO* | 61083-62375 | Type IV prepilin cluster |
| *pilP* | 62347-62796 | Type IV prepilin cluster |
| *pilQ* | 62831-64381 | Type IV prepilin cluster, ATP-binding protein |
| *pilR* | 64397-65491 | Type IV prepilin cluster, integral membrane protein |
| *pilS* | 65499-66113 | Type IV prepilin cluster, prepilin |
| *pilT* | 66123-66683 | Type IV prepilin cluster |
| *pilU* | 66641-67294 | Type IV prepilin cluster, prepilin peptidase |
| *pilV* | 67312-68397 | Type IV prepilin cluster |
| *rci* | 70346-71497 | Shufflon-specific DNA recombinase |
| *traE* | 71687-72511 | F pilus assembly |
| *traF* | 72597-73799 | F pilus assembly |
| *traG* | 73796-74377 | F pilus assembly |
| *traH* | 74832-75287 | F pilus assembly |
| *traI* | 75221-76036 | DNA elicase |
| *traJ* | 76108-77256 | ATP-binding protein |
| *nuc* | 77171-77536 | nuclease |
| *traK* | 77253-77543 | F pilus assembly |
| *sogL* | 78097-81861 | DNA primase |
| *traL* | 81981-82328 | F pilus assembly |
| *traM* | 82307-82996 | Mating signal |
| *traN* | 83019-83999 | Aggregate stability |
| *traO* | 83933-85219 | Hypothetical protein |
| *traP* | 85300-86001 | Conjugal transfer protein |
| *traQ* | 86004-86528 | Conjugal transfer protein |
| *traR* | 86585-86989 | Hypothetical protein |
| *traS* | 87029-87214 | Surface exclusion |
| *traT* | 87219-88016 | Surface exclusion |
| *traU* | 88022-91063 | F pilus assembly |
| *traV* | 91147-91773 | F pilus assembly |
| *traW* | 91739-92939 | F pilus assembly |
