# Supplementary material for: Complete Nucleotide Sequence of CTX-M-15-Plasmids from Clinical Escherichia coli Isolates: Insertional Events of Transposons and Insertion Sequences
Source: PLoS One. 2010 Jun 18;5(6):e11202. doi: 10.1371/journal.pone.0011202 (PMC2887853; doi:10.1371/journal.pone.0011202)
Supplement: Table S2 — (0.11 MB DOC) [file pone.0011202.s002.doc]

**Table S2.** ORFs identified in EC_B24 (73801 bp).

| **Open reading frame (ORF)** | **Position (bp)** | **Protein function** |
| --- | --- | --- |
| *traD* | Compl. 1-2268 | type IV secretion-like conjugative transfer system coupling protein |
| *traT* | Compl. 2494-3249 | conjugal transfer surface exclusion protein |
| *traS* | Compl. 3239-3249 | surface exclusion protein |
| *traG* | Compl. 3745-6570 | conjugal transfer mating pair stabilization protein |
| *traH* | Compl. 6567-7943 | Conjugal transfer protein |
| *trbJ* | Compl. 7940-8383 | Conjugal transfer protein |
| *trbB* | Compl. 8232-8777 | Conjugal transfer protein |
| *traQ* | Compl. 8764-9048 | Conjugal transfer protein |
| *trbA* | Compl. 9167-9514 | type IV secretion-like conjugative transfer system |
| *traF* | Compl. 9530-10321 | F pilus assembly |
| *trbE* | Compl. 10266-10526 | Conjugal transfer protein |
| *traN* | Compl. 10550-12358 | type IV secretion-like conjugative transfer system mating-pair stabilization protein |
| *trbC* | Compl. 12355-13020 | conjugal transfer protein |
| *yfdA* | Compl. 13506-14003 | Hypothetical protein |
| *traU* | Compl. 13990-15084 | F pilus assembly |
| *traW* | Compl. 14979-15725 | F pilus assembly |
| *trbI* | Compl. 15608-16009 | conjugal transfer protein |
| *traC* | Compl. 15991-18624 | F pilus assembly |
| *traR* | Compl. 18778-18999 | Hypothetical protein |
| *traV* | Compl. 19134-19649 | F pilus assembly |
| *trbG* | Compl. 19646-19912 | conjugal transfer protein |
| *trbD* | Compl. 19890-20300 | conjugal transfer protein |
| *traP* | Compl. 20197-20823 | Conjugal transfer protein |
| *traB* | Compl. 20773-22201 | F pilus assembly |
| *traK* | Compl. 22198-22947 | F pilus assembly |
| *traE* | Compl. 22916-23068 | F pilus assembly |
| *yubO* | 23103-23486 | Hypothetical protein |
| *O2R_53* | Compl. 23466-23711 | Hypothetical protein |
| *orf63* | 23785-24009 | Hypothetical protein |
| *mok* | Compl. 24367-24585 | Modulator of Hok protein, Mok |
| *hok* | Compl. 24363-24608 | Post-seggregational killing protein |
| *psiA* | Compl. 24801-25520 | Plasmid SOS inhibition protein A |
| *psiB* | Compl. 25517-26023 | Plasmid SOS inhibition protein B |
| *parB* | Compl. 26020-28080 | ParB-like partitioning protein |
| *ssb* | Compl. 28344-28988 | Single-stranded DNA binding protein |
| *O2R_63* | Compl. 28909-29154 | Hypothetical protein |
| *O2R_65* | 29253-29519 | Hypothetical protein |
| *ydcA* | Compl. 29741-30355 | Hypothetical protein |
| *ydbA* | Compl. 30352-31713 | Hypothetical protein |
| *ydaB* | Compl. 31765-31995 | Hypothetical protein |
| *orf60* | Compl. 32426-32824 | Hypothetical protein |
| *yffA* | Compl. 33033-33290 | Hypothetical protein |
| *ycgC* | Compl. 33221-33643 | Hypothetical protein |
| *klcA* | Compl. 33690-34118 | Antirestriction protein KlcA |
| *tnpA* | 34569-35687 | Transposase of IS186 |
| *ychA* | Compl. 35880-36650 | Hypothetical protein |
| *yubD* | Compl. 37362-38045 | Putative methylase |
| *parM* | 38562-39524 | Plasmid seggregation protein |
| *stbB* | 39524-39877 | Stable plasmid inheritance protein |
| *cba* | 40954-42516 | Colicin-B activity protein |
| *cbi* | Compl. 42534-43088 | Colicin-B immunity protein |
| *cma* | 43277-44119 | Colicin-M activity protein |
| *cmi* | Compl. 44169-44615 | Colicin-M immunity protein |
| *tnpR* | Compl. 44685-45630 | resolvase |
| *atp* | 46387-49107 | ATPase involved in DNA repair |
| *bla*_TEM-33_ | Compl. 50305-51165 | Beta-lactamase TEM-33 precursor |
| *tnpR* | Compl. 51348-52007 | Tn3 resolvase |
| *tnpA* | 52104-52363 | Tn3 transposase (part 1) |
| IS*Ecp1* | 52401-53726 | Transposase |
| *bla*_CTX-M-15_ | 53862-54800 | Beta-lactamase CTX-M-15 precursor |
| *tnpA* | 55193-58045 | Tn3 transposase (part 2) |
| *yacC* | Compl. 59732-60058 | Hypothetical protein |
| *yacB* | Compl. 60077-60355 | Hypothetical protein |
| *yacA* | Compl. 60355-60621 | Hypothetical protein |
| *tnpA* | 60716-61774 | Transposase of IS100 |
| *tnpA* | 61756-62553 | transposase |
| *repA4* | Compl. 62369-62645 | Regulator of *repA1* expression, FII replicon |
| *repA1* | Compl.63499-64365 | Replication initiation protein RepA1 of FII replicon |
| *repA3* | Compl.64369-64560 | Regulator of *repA1* expression, FII replicon |
| *repA2* | Compl. 64648-64905 | Negative regulator of *repA1* expression, FII replicon |
| *rmo* | Compl. 65777-66013 | Modulator of gene expression |
| *yigB* | Compl. 66032-66559 | Hypothetical protein |
| *yigA* | Compl. 66738-66947 | Hypothetical protein |
| *finO* | Compl. 67085-67642 | Conjugative transfer, regulation |
| *traX* | Compl. 67700-68511 | F pilin acetylation |
| *traI* | Compl. 68533-73801 | type IV secretion-like conjugative transfer relaxase protein |
