# Supplementary material for: Complete Nucleotide Sequence of CTX-M-15-Plasmids from Clinical Escherichia coli Isolates: Insertional Events of Transposons and Insertion Sequences
Source: PLoS One. 2010 Jun 18;5(6):e11202. doi: 10.1371/journal.pone.0011202 (PMC2887853; doi:10.1371/journal.pone.0011202)
Supplement: Table S3 — (0.17 MB DOC) [file pone.0011202.s003.doc]

**Table S3.** ORFs identified in EC_L8 (118525 bp).

| **Open reading frame (ORF)** | **Position (bp)** | **Protein function** |
| --- | --- | --- |
| *yjhH* | 1-891 | Dihydrodipicolinate synthase |
| *tdcF* | 913-1296 | Translation initiation inhibitor |
| *kdgT* | 1326-2294 | Putative 2-keto-3-deoxygluconate permease |
| *yfaX* | Compl. 2340-3098 | Putative HTH-type transcriptional regulator |
| *IPF_103* | 3697-3981 | Hypothetical protein |
| *IPF_101* | 3978-4256 | Hypothetical protein |
| *IPF_100* | 4293-4655 | Hypothetical protein |
| *IPF_99* | Compl. 5096-6025 | Hypothetical protein |
| *IPF_393* | 6512-6733 | Hypothetical protein |
| *insA* | 7108-7383 | Insertion element IS1 protein InsA |
| *insB* | 7302-7805 | Insertion element IS1 protein InsB |
| *xcv* | Compl. 8059-9081 | Hypothetical protein |
| *vagD* | 10625-11380 | Virulence associated gene D |
| *vagC* | Compl. 11119-11523 | Virulence associated gene C |
| *pcar* | 11670-15524 | Hypothetical protein |
| *vagD* | Compl. 15569-15985 | Virulence associated gene D |
| *vagC* | Compl. 15985-16212 | Virulence associated gene C |
| *BASY0020* | 16477-16977 | Hypothetical protein |
| *BASY0021* | 16981-17763 | Hypothetical protein |
| *tnpA* | Compl. 17974-19602 | Transposase of IS66, ORF3 |
| *tnpA* | Compl. 19618-19968 | Transposase of IS66, ORF2 |
| *tnpA* | Compl. 19965-20408 | Transposase of IS66, ORF1 |
| *BASY0022* | 20500-21620 | Hypothetical protein |
| *ccdA* | 22695-22913 | Plasmid maintenance protein, antitoxin component |
| *ccdB* | 22888-23220 | Plasmid maintenance protein, toxin component |
| *resD* | 23221-24027 | Site-specific resolvase that cleaves at the rfsF site |
| *repE* | 24801-25556 | Replication initiation protein of the FIA replicon |
| *orf1176* | 26135-27310 | Hypothetical protein |
| *sopB* | 27307-28281 | Plasmid partitioning protein |
| *yccB* | 28872-29963 | Hypothetical protein |
| *yhdJ* | 30348-31031 | DNA methylase |
| *orf73* | 31032-31253 | Hypothetical protein |
| *IPF_24* | 31698-32528 | Hypothetical protein |
| *O2R_74* | Compl. 32644-32814 | Hypothetical protein |
| *klcA* | 32942-33370 | Antirestriction protein |
| *ycjA* | 33417-33839 | Hypothetical protein |
| *ydaA* | 34236-34634 | Hypothetical protein |
| *ydaB* | 35059-35286 | Hypothetical protein |
| *ydbA* | 35344-36705 | Hypothetical protein |
| *orf59* | 36752-37294 | Hypothetical protein |
| *orf63* | Compl. 37612-37836 | Hypothetical protein |
| *ssb* | 38073-38705 | Single-stranded DNA-binding protein |
| *parB* | 38975-41011 | ParB-like partitioning protein |
| *psiB* | 41063-41500 | Plasmid SOS inhibition protein B |
| *psiA* | 41497-42216 | Plasmid SOS inhibition protein A |
| *hok* | 42409-42624 | Post-seggregational killing protein |
| *mok* | Compl. 42228-42458 | Modulator of Hok protein, Mok |
| *orf 63* | Compl. 43008-43232 | Hypothetical protein |
| *orf61* | Compl. 43276-43509 | Hypothetical protein |
| *yubP* | 44921-45073 | Hypothetical protein |
| *gene X* | Compl. 45099-45776 | X-polypeptide, transglycosylation |
| *traM* | 46026-46415 | Mating signal |
| *traJ* | 46549-47295 | Regulation |
| *traY* | 47389-47616 | Ori T nicking |
| *traA* | 47626-48012 | F pilin subunit |
| *traL* | 48014-48328 | F pilin assembly |
| *traE* | 48350-48916 | F pilin assembly |
| *traK* | 48882-49631 | F pilin assembly |
| *traB* | 49628-51058 | F pilin assembly |
| *traP* | 51006-51632 | Conjugal transfer protein |
| *trbD* | 51529-51939 | Conjugal transfer protein |
| *trbG* | 51917-52183 | Conjugal transfer protein |
| *traV* | 52130-52692 | F pilin assembly |
| *traR* | 52830-53051 | Conjugal transfer protein |
| *traC* | 53205-55835 | F pilin assembly |
| *trbI* | 55820-56221 | Conjugal transfer protein |
| *traW* | 56104-56850 | F pilin assembly |
| *traU* | 56820-57839 | F pilin assembly |
| *yfdA* | 57863-58174 | Hypothetical protein |
| *trbC* | 58171-58821 | F pilin assembly |
| *traN* | 58818-60626 | Type IV secretion-like conjugative transfer system mating-pair stabilization protein |
| *trbE* | 60650-60910 | Conjugal transfer protein |
| *traF* | 60855-61643 | F pilin assembly |
| *trbA* | 61662-62000 | Conjugal transfer protein |
| *traQ* | 62127-62411 | Conjugal transfer protein |
| *trbB* | 62398-62943 | F pilin assembly periplasmic protein |
| *trbJ* | 62792-63214 | Conjugal transfer protein |
| *trbF* | 63162-63587 | Conjugal transfer protein |
| *traH* | 63568-64947 | F pilin assembly |
| *traG* | 64944-67763 | Type IV secretion-like conjugative transfer system protein |
| *traS* | 67782-68279 | Conjugal transfer protein |
| *traT* | 68161-69042 | Conjugal transfer surface exclusion protein |
| *traD* | 69268-71520 | Coupling protein |
| *traI* | 71517-76790 | Conjugal transfer nickase/helicase |
| *traX* | 76339-77556 | F pilin acetylation protein |
| *finO* | 78554-79138 | Fertility inhibition protein |
| *yigA* | 79228-79479 | Hypothetical protein |
| *yigB* | 79634-80364 | Hypothetical protein |
| *hhA* | 80201-80440 | Hypothetical protein |
| *yihA* | 80478-81068 | Hypothetical protein |
| *repA2* | 81308-81568 | Negative regulator of *repA1* expression, FII replicon |
| *repA3* | 81653-81847 | Regulator of *repA1* expression, FII replicon |
| *repA1* | 81848-82717 | Replication initiation protein RepA1 of FII replicon |
| *repA4* | 83005-83466 | Regulator of *repA1* expression, FII replicon |
| *bla*_TEM-1_ | Compl. 83743-84603 | Beta-lactamase TEM-1 precursor |
| *tnpR* | Compl. 84786-85343 | Tn3 resolvase |
| *tnpA* | 85342-85722 | Tn3 transposase (part 1) |
| IS*Ecp1* | 85904-87169 | Transposase |
| *bla*_CTX-M-15_ | 87365-88300 | Beta-lactamase CTX-M-15 precursor |
| *tnpA* | 88696-91488 | Tn3 transposase (part 2) |
| *tir* | 91587-92240 | Transfer inhibition protein |
| *pemI* | 92333-92590 | Stable plasmid inheritance, antitoxin |
| *pemK* | 92460-92924 | Stable plasmid inheritance, toxin |
| *tnpA* | Compl. 93061-96012 | Transposase of Tn501 |
| *tnpR* | 95981-96658 | Resolvase of Tn501 |
| *tnpA* | Compl. 96681-97500 | Transposase of Tn1721, truncated |
| *pecM* | 97965-98841 | Hypothetical transmembrane protein |
| *tetA* | Compl. 98881-100173 | Tetracycline efflux protein |
| *tetR* | 100156-100836 | Tetracycline repressor protein |
| *tnpA* | Compl. 101155-103161 | Transposase of Tn1721, truncated |
| *tnpA* | 102616-103338 | Transposase of IS26 |
| *aac6’-lb-cr* | 103344-104036 | Aminoglycoside N(6’)-acetyltransferase |
| *blaOXA-1* | 104122-104997 | Beta-lactamase OXA-1 precursor |
| *catB4* | 105135-105683 | Chloramphenicol acetyl transferase |
| *tnpA* | Compl. 105632-105979 | Transposase of IS26 |
| *yigB* | 106326-106571 | Oxidoreductase |
| *yigB* | 106599-106829 | Oxidoreductase |
| *tnpA* | 106950-107480 | Transposase of IS26 |
| *catB4* | Compl. 107617-108165 | Chloramphenicol acetyl transferase |
| *blaOXA-1* | Compl. 108293-109003 | Beta-lactamase OXA-1 precursor, truncated |
| *aac6’-lb-cr* | Compl. 109262-109905 | Aminoglycoside N(6’)-acetyltransferase (partial) |
| *tnpA* | Compl. 109906-110725 | Transposase of IS26 |
| *orfB* | 110983-111453 | putative transposase OrfB protein of insertion sequence IS629 |
| *orfB* | 111377-111883 | putative transposase OrfB protein of insertion sequence IS629 |
| *tnpA* | 111926-112138 | Hypothetical protein |
| *tnpA* | 112158-112523 | Transposase of IS4 |
| *ugpB* | Compl. 112582-114057 | Putative ABC transporter permease protein |
| *ugpC* | Compl. 113885-115126 | Putative ABC transporter ATP-binding protein |
| *icc* | Compl. 114964-115788 | Phosphodiesterase |
| *araQ* | Compl. 115799-116686 | Putative ABC transporter permease protein |
| *ugpA* | Compl. 116676-117563 | Putative ABC transporter permease protein |
| *yigB* | Compl. 117685-117915 | Oxidoreductase |
| *yigB* | Compl. 117943-118188 | Oxidoreductase |
