# Supplementary material for: Complete Nucleotide Sequence of CTX-M-15-Plasmids from Clinical Escherichia coli Isolates: Insertional Events of Transposons and Insertion Sequences
Source: PLoS One. 2010 Jun 18;5(6):e11202. doi: 10.1371/journal.pone.0011202 (PMC2887853; doi:10.1371/journal.pone.0011202)
Supplement: Table S4 — (0.20 MB DOC) [file pone.0011202.s004.doc]

**Table S4.** ORFs identified in EC_L46 (144871 bp).

| **Open reading frame (ORF)** | **Position (bp)** | **Protein function** |
| --- | --- | --- |
| *tnpA* | Compl. 561-1380 | Transposase of IS26 |
| *orfB* | 1638-2108 | putative transposase OrfB protein of insertion sequence IS629 |
| *orfB* | 2032-2538 | putative transposase OrfB protein of insertion sequence IS629 |
| *tnpA* | 2558-2790 | Hypothetical protein |
| *tnpA* | 2813-3178 | Transposase of IS4 |
| *ugpB* | Compl. 3237-4712 | Putative ABC transporter permease protein |
| *ugpC* | Compl. 4553-5781 | Putative ABC transporter ATP-binding protein |
| *icc* | Compl. 5619-6443 | Phosphodiesterase |
| *araQ* | Compl. 6454-7341 | Putative ABC transporter permease protein |
| *ugpA* | Compl. 7331-8218 | Putative ABC transporter permease protein |
| *yigB* | Compl. 8334-8570 | Oxidoreductase |
| *yigB* | Compl. 8598-8843 | Oxidoreductase |
| *yjhH* | 9181-10071 | Dihydrodipicolinate synthase |
| *tdcF* | 10093-10476 | Translation initiation inhibitor |
| *kdgT* | 10506-11474 | Putative 2-keto-3-deoxygluconate permease |
| *yfaX* | Compl. 11522-12278 | Putative HTH-type transcriptional regulator |
| *IPF_103* | 12877-13161 | Hypothetical protein |
| *IPF_101* | 13158-13436 | Hypothetical protein |
| *IPF_100* | 13473-13835 | Hypothetical protein |
| *IPF_99* | Compl. 14276-15205 | Hypothetical protein |
| *IPF_393* | 15692-15913 | Hypothetical protein |
| *insA* | 16288-16563 | Insertion element IS1 protein InsA |
| *insB* | 16482-16985 | Insertion element IS1 protein InsB |
| *xcv* | Compl. 17239-18261 | Hypothetical protein |
| *vagD* | 19805-20560 | Virulence associated gene D |
| *vagC* | Compl. 20299-20703 | Virulence associated gene C |
| *pcar* | 20850-24705 | Hypothetical protein |
| *vagD* | Compl. 24749-25165 | Virulence associated gene D |
| *vagC* | Compl. 25162-25392 | Virulence associated gene C |
| *BASY0020* | 25657-26157 | Hypothetical protein |
| *BASY0021* | 26161-26943 | Hypothetical protein |
| *tnpA* | Compl. 27154-28782 | Transposase of IS66, ORF3 |
| *tnpA* | Compl. 28798-29148 | Transposase of IS66, ORF2 |
| *tnpA* | Compl. 29145-29588 | Transposase of IS66, ORF1 |
| *BASY0022* | 29680-30800 | Hypothetical protein |
| *ccdA* | 31875-32093 | Plasmid maintenance protein, antitoxin component |
| *ccdB* | 32068-32400 | Plasmid maintenance protein, toxin component |
| *resD* | 32401-33207 | Site-specific resolvase that cleaves at the rfsF site |
| *repE* | 33981-34736 | Replication initiation protein of the FIA replicon |
| *orf1176* | 35315-36490 | Hypothetical protein |
| *sopB* | 36487-37461 | Plasmid partitioning protein |
| *yccB* | 38052-39143 | Hypothetical protein |
| *yhdJ* | 39528-40211 | DNA methylase |
| *orf73* | 40212-40433 | Hypothetical protein |
| *IPF_24* | 40878-41708 | Hypothetical protein |
| *O2R_74* | Compl. 41824-41994 | Hypothetical protein |
| *klcA* | 42122-42550 | Antirestriction protein |
| *ycjA* | 42597-43019 | Hypothetical protein |
| *ydaA* | 43416-43814 | Hypothetical protein |
| *ydaB* | 44239-44466 | Hypothetical protein |
| *ydbA* | 44524-45885 | Hypothetical protein |
| *orf59* | 45932-46474 | Hypothetical protein |
| *orf63* | Compl. 46792-47016 | Hypothetical protein |
| *ssb* | 47253-47885 | Single-stranded DNA-binding protein |
| *parB* | 48155-50191 | ParB-like partitioning protein |
| *psiB* | 50243-50680 | Plasmid SOS inhibition protein B |
| *psiA* | 50677-51396 | Plasmid SOS inhibition protein A |
| *hok* | 51589-51804 | Post-seggregational killing protein |
| *mok* | Compl. 51408-51638 | Modulator of Hok protein, Mok |
| *orf 63* | Compl. 52188-52412 | Hypothetical protein |
| *orf61* | Compl. 52456-52689 | Hypothetical protein |
| *yubP* | 54101-54253 | Hypothetical protein |
| *gene X* | Compl. 54279-54956 | X-polypeptide, transglycosylation |
| *traM* | 55209-55595 | Mating signal |
| *traJ* | 55729-56475 | Regulation |
| *traY* | 56569-56796 | Ori T nicking |
| *traA* | 56806-57192 | F pilin subunit |
| *traL* | 57194-57508 | F pilin assembly |
| *traE* | 57530-58096 | F pilin assembly |
| *traK* | 58062-58811 | F pilin assembly |
| *traB* | 58808-60238 | F pilin assembly |
| *traP* | 60187-60809 | Conjugal transfer protein |
| *trbD* | 60707-61116 | Conjugal transfer protein |
| *trbG* | 61097-61363 | Conjugal transfer protein |
| *traV* | 61360-61875 | F pilin assembly |
| *traR* | 62010-62231 | Conjugal transfer protein |
| *traC* | 62385-65018 | F pilin assembly |
| *trbI* | 65000-65401 | Conjugal transfer protein |
| *traW* | 65284-66030 | F pilin assembly |
| *traU* | 66000-67019 | F pilin assembly |
| *orfF* | 67043-67354 | Hypothetical protein |
| *trbC* | 67351-68001 | F pilin assembly |
| *traN* | 67998-69806 | Type IV secretion-like conjugative transfer system mating-pair stabilization protein |
| *trbE* | 69830-70090 | Conjugal transfer protein |
| *traF* | 70035-70823 | F pilin assembly |
| *trbA* | 70842-71180 | Conjugal transfer protein |
| *traQ* | 71307-71591 | Conjugal transfer protein |
| *trbB* | 71578-72123 | F pilin assembly periplasmic protein |
| *trbJ* | 71972-72394 | Conjugal transfer protein |
| *trbF* | 72342-72767 | Conjugal transfer protein |
| *traH* | 72748-73281 | F pilin assembly, truncated |
| *tnpA* | 73313-74035 | Transposase of IS26 |
| *tnpR* | Compl. 74361-75020 | Tn3 reolvase |
| *tnpA* | 75019-75279 | Tn3 transposase (partial) |
| *tnpA* | Compl. 75281-76000 | Transposase of IS26 |
| IS*Ecp1* | 76307-77572 | Transposase |
| *bla*_CTX-M-15_ | 77768-78703 | Beta-lactamase CTX-M-15 precursor |
| *tnpA* | 79099-81888 | Tn3 transposase (part 2) |
| *tir* | 81990-82643 | Transfer inhibition protein |
| *pemI* | 82736-82993 | Stable plasmid inheritance, antitoxin |
| *pemK* | 82863-83327 | Stable plasmid inheritance, toxin |
| *tnpA* | Compl.83464-86415 | Transposase of Tn501 |
| *tnpR* | 86384-87061 | Resolvase of Tn501 |
| *tnpA* | 87645-88367 | Transposase of IS26 |
| *orf2* | Compl. 89284-89595 | Hypothetical protein |
| *kikA* | Compl. 89631-90017 | Hypothetical protein |
| *traL* | 90716-91453 | Conjugal transfer protein |
| *korA* | 91450-91743 | Hypothetical protein |
| *traM* | 91753-92046 | Conjugal transfer protein |
| *traA* | 92069-92413 | Hypothetical protein |
| *traB* | 92951-93265 | Hypothetical protein |
| *traC* | 94980-95744 | Hypothetical protein |
| *eep* | 95665-95979 | Entry exclusion protein |
| *traD* | 95995-97035 | Conjugal transfer protein |
| *traN* | 97103-97264 | Conjugal transfer protein |
| *traE* | 97233-97952 | Conjugal transfer protein |
| *traO* | 97927-98847 | Type IV conjugative transfer system protein |
| *traF* | 98844-100007 | Type IV conjugative transfer system protein |
| *traG* | 100046-101044 | Type IV secretion-like conjugative transfer system protein |
| *orf8* | Compl. 101750-102136 | Hypothetical protein |
| *orf7* | Compl. 102319-102894 | Hypothetical protein |
| *fipA* | Compl. 103417-103818 | Fertility inhibition protein |
| *traI* | Compl. 103815-107054 | Conjugal transfer nickase/helicase |
| *traJ* | Compl. 107051-108628 | Regulation |
| *traK* | Compl. 108582-108998 | F pilin assembly |
| *stbA* | 109566-109952 | Hypothetical protein |
| *stbB* | 109961-110674 | Hypothetical protein |
| *stbC* | 110677-111047 | Hypothetical protein |
| *orfD* | 111341-111611 | Hypothetical protein |
| *ccgAI* | Compl. 112054-112233 | Hypothetical protein |
| *repA* | Compl. 113032-113740 | Replication initiation protein |
| *orf46* | Compl. 113733-113948 | Hypothetical protein |
| *ardK* | 114188-114649 | Antirestriction protein |
| *mpr* | Compl. 114664-115494 | Putative metalloprotease |
| *mucB* | Compl. 115606-116910 | DNA-directed DNA polymerase activity protein |
| *mucA* | Compl. 116859-117305 | DNA-directed DNA polymerase activity protein |
| *ardB* | 117712-118140 | Antirestriction protein |
| *ardR* | 118177-118602 | Antirestriction protein |
| *ccgEIII* | 118612-118851 | Hypothetical protein |
| *tnpA* | 119825-120547 | Transposase of IS26 |
| *traH* | 120553-121413 | F pilin assembly, truncated |
| *traG* | 121464-124286 | Type IV secretion-like conjugative transfer system protein |
| *traS* | 124302-124802 | Conjugal transfer protein |
| *traT* | 124681-125508 | Conjugal transfer surface exclusion protein |
| *traD* | 125788-127992 | Coupling protein |
| *traI* | 128046-133313 | Conjugal transfer nickase/helicase |
| *traX* | 132868-133770 | F pilin acetylation protein |
| *finO* | 135083-135640 | Fertility inhibition protein |
| *yigA* | 135757-135966 | Hypothetical protein |
| *yigB* | 136163-136696 | Hypothetical protein |
| *hhA* | 136730-136936 | Hypothetical protein |
| *yihA* | 137007-137597 | Hypothetical protein |
| *repA2* | 137837-138097 | Negative regulator of *repA1* expression, FII replicon |
| *repA3* | 138377-139231 | Regulator of *repA1* expression, FII replicon |
| *repA1* | 139534-139917 | Replication initiation protein RepA1 of FII replicon |
| *repA4* | 140005-140187 | Regulator of *repA1* expression, FII replicon |
| *bla*_TEM-1_ | Compl. 140197-141054 | Beta-lactamase TEM-1 precursor |
| *tnpA* | 141055-141774 | Transposase of IS26 |
| *catB4* | Compl.141769-142313 | Chloramphenicol acetyl transferase |
| *blaOXA-1* | Compl. 142452-143320 | Beta-lactamase OXA-1 precursor |
| *aac6’-lb-cr* | Compl. 143408-144051 | Aminoglycoside N(6’)-acetyltransferase |
| *tnpA* | Compl. 144052-144871 | Transposase of IS26 |
